# Self-reported anticipated compliance with physician advice to stay home during pandemic (H1N1) 2009: Results from the 2009 Queensland Social Survey

**DOI:** 10.1186/1471-2458-10-138

**Published:** 2010-03-16

**Authors:** Lawrence H Brown, Peter Aitken, Peter A Leggat, Richard Speare

**Affiliations:** 1 Anton Breinl Centre, School of Public Health, Tropical Medicine and Rehabilitation Sciences, James Cook University, Townsville, Queensland, Australia; 2 Department of Emergency Medicine, The Townsville Hospital, Townsville, Queensland, Australia

## Abstract

**Background:**

One strategy available to public health officials during a pandemic is physician recommendations for isolation of infected individuals. This study was undertaken during the height of the Australian pandemic (H1N1) 2009 outbreak to measure self-reported willingness to comply with physician recommendations to stay home for seven days, and to compare responses for the current strain of pandemic influenza, avian influenza, seasonal influenza, and the common cold.

**Methods:**

Data were collected as part of the Queensland Social Survey (QSS) 2009, which consisted of a standardized introduction, 37 demographic questions, and research questions incorporated through a cost-sharing arrangement. Four questions related to respondents' anticipated compliance with a physician's advice to stay home if they had a common cold, seasonal influenza, pandemic (H1N1) 2009 influenza or avian influenza were incorporated into QSS 2009, with responses recorded using a balanced Likert scale ranging from "very unlikely" to "very likely." Discordance between responses for different diseases was analysed using McNemar's test. Associations between demographic variables and anticipated compliance were analysed using Pearson's chi-square or chi-square for linear-by-linear association, and confirmed using multivariate logistic regression; p < 0.05 was used to establish statistical significance.

**Results:**

Self-reported anticipated compliance increased from 59.9% for the common cold to 71.3% for seasonal influenza (p < .001), and to 95.0% for pandemic (H1N1) 2009 influenza and 94.7% for avian influenza (p < 0.001 for both versus seasonal influenza). Anticipated compliance did not differ for pandemic (H1N1) 2009 and avian influenza (p = 0.815). Age and sex were both associated with anticipated compliance in the setting of seasonal influenza and the common cold. Notably, 27.1% of health and community service workers would not comply with physician advice to stay home for seasonal influenza.

**Conclusions:**

Ninety-five percent of people report they would comply with a physicians' advice to stay home for seven days if they are diagnosed with pandemic (H1N1) 2009 or avian influenza, but only 71% can be expected to comply in the setting of seasonal influenza and fewer still can be expected to comply if they are diagnosed with a common cold. Sub-populations that might be worthwhile targets for public health messages aimed at increasing the rate of self-imposed isolation for seasonal influenza include males, younger people, and healthcare workers.

## Background

In late March 2009 an outbreak of a new strain of influenza A (H1N1), swine-origin influenza virus (S-OIV) or "swine flu," was reported in North America [1,2]. This disease quickly spread across the globe, and the World Health Organization declared a pandemic on 11 June 2009 [3]. The first cases of pandemic (H1N1) 2009 influenza in Australia were reported in May 2009, coinciding with the onset of the annual influenza season. As of 01 January 2010, 37,553 cases of pandemic (H1N1) 2009 influenza had been confirmed in Australia, with 191 deaths [4].

At the time the Australian cases peaked, there was no approved vaccine for pandemic (H1N1) 2009 virus; traditional public health measures were critical to containing the outbreak. One strategy available to public health officials is physician recommendations for self-imposed isolation of infected individuals; specifically, to stay home for at least seven days. Such public health measures, however, only work if patients are willing to comply [5-7]. This study was undertaken during the height of the Australian pandemic (H1N1) 2009 outbreak to measure self-reported willingness to comply with physician recommendations to stay home for seven days, and to compare responses for the current strain of pandemic influenza, avian influenza (H5N1), seasonal influenza, and the common cold.

## Methods

Data for this study were collected as part of the Queensland Social Survey (QSS) 2009. QSS is an annual state-wide survey conducted by the Population Research Laboratory (PRL) in CQUniversity Australia's Institute for Health and Social Science Research. Through a cost-sharing arrangement, QSS enables researchers and policy-makers to incorporate questions into the survey.

Queensland is the second largest Australian state by land area, and the third most populous state. QSS uses a computer-assisted telephone interviewing (CATI) system and trained interviewers to randomly sample households across Queensland, including metropolitan Brisbane (South East Queensland) and the rest of the state (Other Queensland). To ensure equal representation of males and females, households are randomly pre-determined to provide a male or female respondent; if a person of that sex is not available then the household is not included in the survey.

QSS 2009 consisted of a standardized introduction, specific questions incorporated by researchers and the University, and 37 demographic questions. The questions were pilot tested by trained interviewers in 92 randomly-selected households, with modifications to the questions guided by both responses from the pilot study subjects and feedback from the interviewers. Final interviewing was conducted between 20 July 2009 and 19 August 2009, between the hours of 10:30 am to 2:30 pm and 4:30 pm to 8:30 pm on weekdays, and between the hours of 11:00 am and 4:00 pm on weekends.

Four questions related to respondents' anticipated compliance with a physician's advice to stay home if they had a viral respiratory illness were incorporated into QSS 2009. The four questions were:

• If you had a common cold and your doctor recommended that you stay home for at least seven days so as not to infect anyone else, how likely are you to do so?

• If you had the regular flu, but not swine or bird flu, and your doctor recommended that you stay home for at least seven days so as not to infect anyone else, how likely are you to do so?

• If you had the swine flu and your doctor recommended that you stay home for at least seven days so as not to infect anyone else, how likely are you to do so?

• If you had the avian or bird flu and your doctor recommended that you stay home for at least seven days so as not to infect anyone else, how likely are you to do so?

Responses were recorded using a 4-point Likert scale ranging from "very unlikely" to "very likely." Responses were subsequently dichotomized as "yes" (very likely or likely) and "no" (very unlikely or unlikely) and cross-tabulated in a 2 × 2 table. Because the data are essentially repeated measures of likelihood to comply under different circumstances, discordance between responses for the different diseases was analysed using McNemar's test. Bivariate associations between relevant demographic variables and anticipated compliance were analysed using chi-square or Fisher's exact test; where demographic variables were recorded as ordinal data, analyses utilizing chi-square for linear-by-linear association were conducted to identify any significant trend effects. Subsequently, multivariate logistic regression was conducted to identify covariates and interaction effects, and to adjust for confounding. Each variable was entered into or removed from the logistic regression model using both forward and backward methods to identify significant covariates, the remaining variables were then individually entered into the model to identify potential confounders. The final model included significant covariates, potential confounders and significant interaction effects. For all analyses, p < 0.05 was used to establish statistical significance; for the multivariate analysis, adjusted odds ratios (AOR) and their 95% confidence intervals (CI) are reported.

QSS 2009 had a target sample size of 1,200 subjects, with 800 subjects from South East Queensland and 400 from Other Queensland; thus the *a priori *estimated sampling error at the 95% confidence level was ± 2.9% overall, ± 3.6% for the South East Queensland sub-sample, and ± 5.1% for the Other Queensland sub-sample.

QSS 2009 was approved by the Human Ethics Review Panel at CQUniversity (H09/06-037) and the incorporation of the influenza-related questions was approved by the Human Research Ethics Committee at James Cook University (H3456).

## Results

QSS 2009 contacted or attempted to contact 3,112 households; 1,536 subjects declined participation, 142 households could not be contacted, and 129 were otherwise ineligible. Thus, the final sample for QSS 2009 included 1,292 respondents; 860 from South East Queensland and 432 from Other Queensland for an overall response rate of 41.5%. The sample was nearly equally divided between males and females (50.2% vs. 49.8%). Younger people (aged 18 - 34 years) were under-represented in the sample and older people (aged > 55 years) were over-represented in the sample, otherwise the demographics of the participants reasonably approximated that of the general population[8] as shown in Table 1.

**Table 1:** Demographic characteristics of the QSS sample and of Queensland, Australia. [8]

	**QSS Sample**	**Queensland**
**Age**		
18-34	13.0%	30.6%
35-44	20.0%	19.6%
45-54	20.3%	18.4%
55+	56.2%	31.4%
		
**Sex**		
Male	50.2%	49.6%
Female	49.8%	50.4%
		
Employment Status*		
Full-time	35.8%	38.1%
Part-time/Casual	19.4%	17.1%
Unemployed	3.2%	2.9%
Other/Not in Labour Force	40.1%	38.2%
		
Household Income*		
$0-26,000	17.3%	18.3%
$26,001-52,000	14.1%	24.1%
$52,001-100,000	20.1%	31.5%
$100,001+	20.3%	14.7%
		
Marital Status*		
Married/Partnered	75.2%	60.2%
Single	24.6%	39.8%

* The Australian Bureau of Statistics uses slightly different categories and thresholds than QSS 2009.

Responses to the four questions concerning anticipated compliance with a physician's advice to stay home are shown in Table 2. Self-reported anticipated compliance increased significantly from 59.9% for the common cold to 71.3% for seasonal influenza (McNemar's test, p < .001), and to 95.0% for pandemic (H1N1) 2009 influenza and 94.7% for avian influenza (McNemar's test, p < 0.001 for both versus seasonal influenza). Anticipated compliance did not differ for pandemic (H1N1) 2009 and avian influenza (McNemar's test, p = 0.815).

**Table 2:** Likelihood of complying with a physician's advice to stay home if diagnosed with a viral respiratory disease.

	**Common Cold**	**Seasonal Influenza**	**Pandemic (H1N1) 2009**	**Avian Influenza**
Very Unlikely	16.5%	9.4%	2.6%	2.6%
Unlikely	22.7%	18.1%	1.5%	1.3%
Likely	28.6%	33.8%	14.0%	13.3%
Very Likely	31.3%	37.5%	81.0%	81.4%
Don't Know	0.7%	0.8%	0.5%	0.9%
No Response	0.2%	0.3%	0.2%	0.4%
"Would Comply"	59.9%	71.3%	95.0%	94.7%
"Would Not Comply"	26.2%	27.5%	4.1%	3.9%

"Would Comply" = (Very Likely + Likely)

"Would Not Comply" = (Very Unlikely + Unlikely)

Bivariate associations between demographic variables and anticipated compliance with a physician's advice to stay home for the four viral diseases are shown in Additional file 1: Table S1. As anticipated compliance in the setting of pandemic (H1N1) 2009 and avian influenza was near universal, there were no significant associations between demographic variables and anticipated compliance. For the common cold and seasonal influenza, however, there were a number of significant associations. Respondents who were male, younger, employed (versus unemployed), and had a higher level of education were less likely to report anticipated compliance with stay home advice for both a common cold and seasonal influenza. Married/partnered people and those who lived in South East Queensland were also less likely to comply with advice to stay home for a common cold. People who lived in urban areas, and people employed in the health and community services sector were more likely than others to comply with advice to stay home for seasonal influenza, although 27.1% of health and community service workers would be unlikely to comply with such advice.

In multivariate analysis, only sex and age remained significantly associated with anticipated compliance, and there was no interaction effect between these two variables. (Additional file 2: Table S2) Females were more likely than males to report anticipated compliance for both the common cold (AOR = 1.650; CI: 1.143-2.381) and seasonal influenza (AOR = 1.911; CI: 1.300-2.811). People age 55 and older were also more likely to report anticipated compliance for both the common cold (AOR = 1.542; CI: 1.002-2.372) and seasonal influenza (AOR = 2.316; CI: 1.431-3.749) when compared to younger respondents.

## Discussion

Nearly every respondent in this study reported they would comply with a doctor's advice to stay home for seven days if they were diagnosed with pandemic (H1N1) 2009 influenza, and the same level of compliance could be expected in the setting of avian influenza. These findings are similar to those that have been previously reported; our study adds data in the context of an actual, rather than hypothetical, pandemic.

Prior to the current pandemic, Eastwood et al read a brief description of a pandemic influenza outbreak analogous to the 1918 Spanish flu to Australian telephone survey participants, and found 97.5% of respondents would stay home for seven to ten days if they were told they might have had contact with the disease [7]. Similarly, Barr et al[9] reported 85% of Australians would be at least moderately willing to isolate themselves from others during an influenza pandemic. Blendon et al[10] reported 94% of Americans would comply if they contracted a pandemic influenza and public health officials recommended they stay at home for seven to ten days. In a more recent survey from June of 2009, Blendon et al[11] identified 236 respondents who reported that they themselves or someone in their household had experienced flu-like symptoms, and 75% of those with symptoms had stayed home. Other studies have also found support for explicit government action to contain pandemic influenza, including "encouraging" people to work from home, and quarantining infected individuals [5,12]. Interestingly, DiGiovanni et al[13] reported that compliance with quarantine measures during the 2003 severe acute respiratory syndrome (SARS) outbreak in Toronto, Canada was affected more by compliance monitoring, fighting boredom and stress, and minimizing stigmatization than with any actual threat of enforcement.

From a public health planning perspective, the more useful data from this study may be that regarding the level of compliance with stay at home advice that can be anticipated for seasonal influenza, and the relative lack of compliance that can be expected for the common cold. Seasonal influenza is a more common disease, each year leading to approximately 18,000 hospitalizations and costing around $115 million in Australia; the burden in the United States is much greater with the direct costs of influenza-related medical care exceeding $10 billion [14]. Yet, these data confirm that people do not view seasonal influenza with the same level of concern as pandemic strains of influenza. While it is encouraging that respondents appear to differentiate between seasonal influenza and the common cold, the questions in this survey presumed a physician diagnosis. Large numbers of people do not seek medical care for mild to moderate respiratory illness, and it is not practical to expect lay people to reliably differentiate between a common cold and influenza. Public health efforts to encourage people to self-isolate for influenza-related illnesses may be more successful if they target symptoms (i.e., "cough and fever") rather than specific diagnoses.

This study did find some significant associations between demographic characteristics and likelihood to comply with stay at home advice for seasonal influenza that might be useful for targeting public health efforts to increase compliance. Males were less likely to report anticipated compliance with stay home advice for both a common cold and seasonal influenza, and this is consistent with other studies from Australia [7,9]. Males have also previously been reported to feel less susceptible than females do to pandemic influenza,[5] although this study found no differences between males and females for anticipated compliance in the setting of pandemic (H1N1) 2009 or avian influenza.

Increasing age was associated with increased anticipated compliance with stay at home advice for both the common cold and seasonal influenza, while increasing education and income were associated with decreased anticipated compliance for both diseases. Although the associations for education and income did not withstand multivariate analysis, the finding is consistent with previous work and both variables were retained as potential confounders in the final logistic regression model. Like males, wealthier and better educated people tend to view themselves as less susceptible to influenza, while older people tend to view themselves as more susceptible [5]. Many influenza-related public health campaigns target older populations; targeting stay at home messages to wealthier and better educated populations might be a novel but worthwhile effort for containing seasonal influenza.

Employed respondents were less likely than unemployed respondents to report anticipated compliance with stay home advice for both a common cold and seasonal influenza. This association, also, did not withstand multivariate analysis, but it is an intuitive finding. Even in the setting of pandemic influenza, many people would have to forgo income in order to stay home [10]. For example, a survey of key decision makers at U.S. businesses found 74% of the businesses provided for paid employee sick leave, but 15% of businesses did not provide for any employee sick leave, whether paid or unpaid [15]. Still, this study found no difference in anticipated compliance rates in the setting of pandemic (H1N1) 2009 or avian influenza. This is consistent with the findings of Barr et al[9] who reported similar rates of "willingness to comply with health protective behaviours" between employed (69.5%, 95%CI: 65.5%-73.5%) and unemployed (71.8%, 95%CI: 67.7%-76.0%) survey respondents in the setting of pandemic influenza. Eastwood et al,[7] however, reported the contrary, finding that employed people who were unable to work from home would be less likely to self-isolate in the setting of pandemic influenza. How closely the level of actual compliance approaches the level of self-reported anticipated compliance may well be affected by issues related to income, financial security, and employer leave policies.

A particularly novel and important finding of this study was that more than one-quarter of health and community service workers reported they would not comply with a physician's advice to stay home if they had seasonal influenza. This may represent a misplaced sense of duty. Previous research has demonstrated that most healthcare workers (HCWs) would not abandon their responsibilities during an influenza pandemic,[16,17] but isolating one's self when one has symptoms or a diagnosis of disease is a different proposition than simply refusing to work. Despite evidence of the efficacy of vaccinating HCWs, [18-23] influenza vaccination rates among HCWs are low,[24] which presents a risk of HCW-to-HCW as well as HCW-to-patient transmission if infected HCWs report to work. Notably, as the 2003 SARS outbreak subsided and precautions were relaxed, a second wave of the disease including 90 cases of nosocomial infections emerged; 42.5% of those nosocomial infections were associated with exposure to an infected HCW. Seventeen nurses contracted SARS, and 12 (70.6%) had worked with a symptomatic co-worker within 10 days of developing symptoms. Indeed, having worked with a symptomatic co-worker was associated with increased risk (RR = 1.88) of an HCW developing the disease [25]. We are not aware of any previous reports measuring anticipated self-isolation among HCWs with influenza. Public health officials and health facility supervisors must impress upon health workers the clinical and ethical importance of protecting both patients and other staff from exposure to employee-borne influenza, including seasonal influenza [26].

This study was limited in that it relied upon a telephone survey to collect data, but telephone surveys have been previously used to gather information regarding public perceptions of risk and willingness to comply with containment strategies for influenza,[5,7,9-12] and even to assess for the prevalence of influenza [27]. The response rate for this survey was 41.5%; while this may indicate some response bias the sample was fairly representative of the general population, and the overall survey was not specific to influenza. That is, there is no reason to suspect that any potential respondent's decision about whether to participate in the survey would be related to their anticipated compliance with a physician's advice to stay home. A more important limitation of the study is that it measured self-reported anticipated behaviour in the context of a physician diagnosis of disease. Actual behaviour may differ, particularly since many individuals with mild to moderate viral respiratory syndromes do not seek physician care. Also, other factors including perceived severity of illness, social norms, and financial considerations could affect compliance. Thus, the rates of anticipated compliance reported by respondents to this survey must be viewed as a best-case scenario, and actual compliance might be lower. Still the results, both in terms of anticipated compliance and associations with demographic factors, are consistent with those of other studies [5,7,9-12]. Finally, early in the Australian pandemic (H1N1) 2009 experience there was a perceived association between international travel and increased risk,[28] but QSS 2009 did not inquire as to respondents' individual travel history or exposure to international travellers.

## Conclusions

Ninety-five percent of people report they would comply with a physicians' advice to stay home for seven days if they are diagnosed with pandemic (H1N1) 2009 or avian influenza, but only 71% can be expected to comply with the same advice in the setting of seasonal influenza and fewer still (60%) can be expected to stay home if they are diagnosed with a common cold. Sub-populations that might be worthwhile targets for public health messages aimed at increasing the rate of self-imposed isolation for seasonal influenza include males and younger people. Notably, more than one-quarter of health and community service workers report that they are unlikely to comply with stay home advice for seasonal influenza; thus they too may be an appropriate (although counter-intuitive) target for influenza-related public health campaigns.

## List of Abbreviations

**CATI**: computer-assisted telephone interviewing; **PRL**: Population Research Laboratory; **QSS**: Queensland Social Survey; **RR**: relative risk; **SARS**: severe acute respiratory syndrome; **S-OIV**: swine origin influenza virus.

## Competing interests

The authors declare that they have no competing interests.

## Authors' contributions

LHB, PA, PAL and RS participated in the development of the research question and the influenza-related questionnaire items for inclusion in QSS 2009. LHB conducted the primary analysis. LHB, PA, PAL and RS participated in the interpretation of the data and the initial drafting of the manuscript. All authors read and approved the final manuscript.

## Pre-publication history

The pre-publication history for this paper can be accessed here:

http://www.biomedcentral.com/1471-2458/10/138/prepub

## Supplementary Material

Additional file 1 **Table S1 - Bivariate associations between demographic variables and anticipated compliance with physician's advice to stay home for seven days for common cold and three strains of influenza**. A table showing the bivariate associations between demographic variables and anticipated compliance. Click here for file

Additional file 2 **Table S2 - Final models and results of the multivariate logistic regression**. A table showing the final models, coefficients, and adjusted odds ratios for the logistic regressions predicting anticipated compliance for the common cold and seasonal influenza. Click here for file
