# Supplementary material for: Self-reported anticipated compliance with physician advice to stay home during pandemic (H1N1) 2009: Results from the 2009 Queensland Social Survey
Source: BMC Public Health. 2010 Mar 16;10:138. doi: 10.1186/1471-2458-10-138 (PMC2847980; doi:10.1186/1471-2458-10-138)
Supplement: Additional file 1 — Table S1 - Bivariate associations between demographic variables and anticipated compliance with physician's advice to stay home for seven days for common cold and three strains of influenza. A table showing the bivariate associations between demographic variables and anticipated compliance. [file 1471-2458-10-138-S1.DOC]

**Table S1: Bivariate associations between demographic variables and anticipated compliance with physician’s advice to stay home for seven days for common cold and three strains of influenza.**

|  | Common  Cold | p | Seasonal  Influenza | p | Pandemic (H1N1) 2009 | p | Avian  Influenza | p |
| --- | --- | --- | --- | --- | --- | --- | --- | --- |
| **Sex** |  |  |  |  |  |  |  |  |
| Male | 54.6% | <0.001 | 64.5% | <0.001 | 94.7% | 0.054 | 95.3% | 0.209 |
| Female | 66.4% |  | 79.8% |  | 96.9% |  | 96.7% |  |
|  |  |  |  |  |  |  |  |  |
| **Age** |  |  |  |  |  |  |  |  |
| 18-34 | 57.1% | <0.001^a^ | 62.3% | <0.001^a^ | 95.2% | 0.701^a^ | 96.4% | 0.360^a^ |
| 35-44 | 47.7% |  | 59.5% |  | 95.3% |  | 96.8% |  |
| 45-54 | 53.5% |  | 64.2% |  | 96.6% |  | 96.2% |  |
| 55+ | 69.9% |  | 83.9% |  | 95.8% |  | 95.4% |  |
|  |  |  |  |  |  |  |  |  |
| **Location** |  |  |  |  |  |  |  |  |
| SE Qld | 58.1% | 0.013 | 71.6% | 0.561 | 95.9% | 0.784 | 96.3% | 0.377 |
| Other Qld | 65.3% |  | 73.2% |  | 95.6% |  | 95.3% |  |
|  |  |  |  |  |  |  |  |  |
| Urban | 60.7% | 0.903 | 73.5% | 0.048 | 95.9% | 0.701 | 96.3% | 0.337 |
| Rural | 60.3% |  | 67.7% |  | 95.4% |  | 95.0% |  |
|  |  |  |  |  |  |  |  |  |
| **Education** |  |  |  |  |  |  |  |  |
| 0-10 years | 72.2% | <0.001^a^ | 78.7% | 0.002^a^ | 96.7% | 0.975^a^ | 97.5% | 0.407^a^ |
| 11-12 years | 60.3% |  | 70.9% |  | 94.7% |  | 94.7% |  |
| 13-14 years | 60.3% |  | 69.5% |  | 93.4% |  | 94.7% |  |
| 15+ years | 51.3% |  | 68.4% |  | 96.5% |  | 96.0% |  |
|  |  |  |  |  |  |  |  |  |

**Table S1 (continued): Bivariate associations between demographic variables and anticipated compliance with physician’s advice to stay home for seven days for common cold and three strains of influenza.**

|  | Common  Cold | p | Seasonal  Influenza | p | Pandemic  (H1N1) 2009 | p | Avian  Influenza | p |
| --- | --- | --- | --- | --- | --- | --- | --- | --- |
| **Income** |  |  |  |  |  |  |  |  |
| $0-26K | 73.0% | <0.001^a^ | 81.9% | <0.001^a^ | 95.1% | 0.867^a^ | 95.4% | 0.651^a^ |
| $26-52K | 66.5% |  | 77.7% |  | 96.1% |  | 95.6% |  |
| $52-100K | 52.5% |  | 64.7% |  | 96.1% |  | 96.1% |  |
| $100K+ | 47.1% |  | 1.4% |  | 95.4% |  | 96.1% |  |
|  |  |  |  |  |  |  |  |  |
| **Marital Status** |  |  |  |  |  |  |  |  |
| Partnered | 58.4% | 0.008 | 71.5% | 0.317 | 95.9% | 0.787 | 96.1% | 0.823 |
| Single | 66.8% |  | 74.4% |  | 95.6% |  | 95.9% |  |
|  |  |  |  |  |  |  |  |  |
| **Employment** |  |  |  |  |  |  |  |  |
| Employed | 50.7% | <0.001^b^ | 63.8% | <0.001^b^ | 96.1% | 0.661^b^ | 96.6% | 0.389^b^ |
| Unemployed | 72.2% |  | 82.7% |  | 95.4% |  | 95.2% |  |
|  |  |  |  |  |  |  |  |  |
| **Field of Work** |  |  |  |  |  |  |  |  |
| Health Field | 57.0% | 0.157 | 72.9% | 0.033 | 96.2% | 0.920 | 97.2% | 0.726 |
| Other Fields | 49.6% |  | 62.2% |  | 96.0% |  | 96.5% |  |

All p values for Pearson’s *X*^2^ except ^a^ = chi-square for linear-by-linear association and ^b^ = Fisher’s Exact Test
