# Supplementary material for: Self-reported anticipated compliance with physician advice to stay home during pandemic (H1N1) 2009: Results from the 2009 Queensland Social Survey
Source: BMC Public Health. 2010 Mar 16;10:138. doi: 10.1186/1471-2458-10-138 (PMC2847980; doi:10.1186/1471-2458-10-138)
Supplement: Additional file 2 — Table S2 - Final models and results of the multivariate logistic regression. A table showing the final models, coefficients, and adjusted odds ratios for the logistic regressions predicting anticipated compliance for the common cold and seasonal influenza. [file 1471-2458-10-138-S2.DOC]

**Table S2: Final models and results of the multivariate logistic regression.**

|  | Common Cold | | | | | Seasonal Influenza | | | | |
| --- | --- | --- | --- | --- | --- | --- | --- | --- | --- | --- |
| **Variable** | B | SE | Sig | AOR | 95%CI | B | SE | Sig | AOR | 95%CI |
| Female | 0.501 | 0.187 | 0.008 | 1.650 | 1.143-2.381 | 0.648 | 0.197 | 0.001 | 1.911 | 1.300-2.811 |
| Age 55+ | 0.433 | 0.220 | 0.049 | 1.542 | 1.002-2.372 | 0.840 | 0.246 | 0.001 | 2.316 | 1.431-3.749 |
| Education > 10 Yrs | -0.159 | 0.254 | 0.532 | 8.53 | 0.518-1.404 | 0.320 | 0.264 | 0.225 | 1.378 | 0.821-2.310 |
| Income > $26K | -- | -- | -- | -- | -- | 0.060 | 0.455 | 0.895 | 1.062 | 0.435-2.590 |
| Income > $52K | -0.443 | 0.228 | 0.052 | 0.64 | 0.411-1.003 | -0.375 | 0.319 | 0.239 | 0.687 | 0.368-1.283 |
| Income > $100K | -- | -- | -- | -- | -- | 0.047 | 0.208 | 0.823 | 1.048 | 0.697-1.576 |
| Health Worker | 0.042 | 0.262 | 0.872 | 1.043 | 0.624-1.744 | 0.145 | 0.282 | 0.608 | 1.156 | 0.665-2.011 |
